# Examining liminality in professional practice, relational identities, and career prospects in resource-constrained health systems: Findings from an empirical study of medical and nurse interns in Kenya

**DOI:** 10.1016/j.socscimed.2024.117226

**Published:** 2024-08-10

**Authors:** Yingxi Zhao, Stephanie Nzekwu, Mwanamvua Boga, Daniel Mbuthia, Jacinta Nzinga, Mike English, Sassy Molyneux, Gerry McGivern

**Affiliations:** a NDM Centre for Global Health Research, Nuffield Department of Medicine, University of Oxford, Oxford, UK; b Warwick Business School, University of Warwick, Warwick, UK; c KEMRI-Wellcome Trust Research Programme, Kilifi, Kenya; d KEMRI-Wellcome Trust Research Programme, Nairobi, Kenya; e King’s Business School, King’s College London, London, UK

**Keywords:** Liminality, Internship, Professional identity, Professional practice, Inter-professional and intra-professional relations, Career transition, Medicine, Nursing, Resource-constrained health systems

## Abstract

We examine new doctors’ and nurses’ experiences of transitioning from training to practising as health professionals, drawing on the concept of *liminality*. Liminality is a stage of ‘in-betweenness’, involving uncertainty and ambiguity as people leave one social context and reintegrate into a new one. Surprisingly little research has explored new health professionals’ experiences of liminality during role and career transitions, particularly in precarious and resource-constrained settings. Drawing on 146 qualitative interviews and seven focus groups, involving 121 new graduate medical doctors and nurses transitioning through internship training in Kenya, we describe three aspects of liminality. First, *liminal professional practice*, where interns realise that best practices learned during medical and nursing schools are often impossible to implement in resource constrained health care settings; instead they learn workarounds and practical norms. Second, *liminal relational identities*, where interns leave behind being students and adopt the identities and responsibilities of qualified professionals within pre-existing professional hierarchies of status and expertise. We explain how these new doctors and graduate nurses negotiate their liminal status, including in relation to more experienced but less qualified professional colleagues. We also discuss how interns cope with liminality due to disappointing and inadequate supervision and role modelling from senior colleagues but then find peer support and their place within their own professions. Finally, we discuss how new doctors and nurses come to terms with the precarity of working in resource constrained health systems, abandon expectations of secure, permanent employment and careers, and accept the realities of *liminal professional careers*. We explain how all three forms of liminality influence professionals’ developing practices, identities, and careers. We call for further studies with a specific liminality lens to explore this critical period in health workers’ careers, to inform policy and practice responding to global transformations in healthcare professions and practice.

## 1 Introduction

“Internship is somewhere between being a student and a medical doctor and then being told that you’re going to be the first contact with the patient, it makes you … feel ‘I’m not good enough for these people’ but then these people are trusting you with their life … that was quite hard.” (Medical doctor, Kenya)

The concept of liminality describes a stage of transition and ‘in-betweenness’, involving ‘separation’ from one social context and ‘reaggregation’ into a new context and social norms (Turner, 1987; van Gennep, 1960). While liminality has been examined in studies of patients’ states of ‘sickness’ and ‘wellness’ (for example Bristowe et al., 2024; Saunders et al., 2018; Schrøder et al., 2021; Trusson et al., 2016), with exceptions of Croft et al. (2015a); Allan et al. (2015); Gordon et al. (2020); Chen and Reay (2021), little research has explored liminality in health professionals’ careers. This is an important oversight, as health professionals face increasingly unstable, ambiguous, and precarious contemporary workplaces (Mbuthia et al., 2021; Söderlund and Borg, 2018), which new health workers may struggle to understand during career transitions, resulting in their later disengagement from the health workforce.

Moreover, the literature examining liminality in health care has overlooked health workers’ experiences in resource-constrained settings within low- and middle-income countries (LMICs), where the majority of the global population live and work. In LMICs, health professionals often experience presents significant barriers to providing good health care (Zhao et al., 2022), potentially intensifying liminal experiences by provoking intense struggles for meaning, identity, and professional status (Mbuthia et al., 2021). Better understanding of how health workers navigate liminal career transitions can help inform training and support processes for them. In this paper, we therefore address the research question: *how do new healthcare professionals experience and respond to liminality while transitioning from training into clinical practice in severely resource-constrained settings?*

To address this question, we analysed qualitative data from three related empirical sub-studies of medical and nursing interns in Kenya. Internships have been identified as a critical liminal period for health care professionals (Bauer et al., 2007), during which they transition from being students to qualified health professionals, experiencing the realities of independent professional practice for the first time.

In Kenya, intern health workers face severe resource constraints, high workloads and disease burdens, poor patient care, preventable deaths, and inadequate accountability and support systems that few envisaged during undergraduate training (Zhao et al., 2023b). Unexpected barriers to providing high-quality health care potentially exacerbate experiences of liminality documented in less constrained contexts. We therefore contribute to the literature on liminality in health care by extending its application into resource-constrained LMIC contexts and using it to examine three dimensions of health professionals’ liminality relating to (1) practice; (2) relational identity; and (3) career prospects.

## 2 Theoretical and empirical background

### 2.1 Liminality

Liminality is a stage of transition and ‘in-betweenness’ that people experience as they separate from one social context and reaggregate into a new one; a stage which often involves confusion, uncertainty, and ambiguity (Beech, 2011; Johnsen and Sørensen, 2015; Söderlund and Borg, 2018; Turner, 1987; van Gennep, 1960). The concept of liminality was first developed in anthropological studies of ‘rites of passage’ (such as marriage and childbirth) marking transitions between stages of life (Turner, 1987; van Gennep, 1960). The concept was subsequently used to explain transitions in careers, professional and organisational settings, requiring people to reconstruct identities and understandings of work contexts (Beech, 2011), accept losing old identities or desired futures (Ybema et al., 2011), and re-oriented towards new roles, identities and relationships (Pratt et al., 2006).

Studies also describe people being stuck in *permanent* liminality or ‘limbo’ between multiple identities (Johnsen and Sørensen, 2015; Söderlund and Borg, 2018; Ybema et al., 2011). For example, temporary workers have been described as stuck between temporary and full-time, regular employment (Garsten, 1999). Bamber et al. (2017) describe ‘teaching-only’ academic staff in Universities as in ‘occupational limbo’. This is conceptually different from liminality in that limbo involves being unable to transition into a desired elevated identity status (being a ‘proper academic’ doing research).

A few studies have explained health workers’ experiences of liminality. For example, Allan et al. (2015) described newly qualified nurses in a liminal transition involving recontextualising previous clinical knowledge. Gordon et al. (2020) describe doctors’ transitions from trainee to qualified doctor as a dynamic liminal phase, during which they feel neither trainee nor trained, involving identity work reconstructing professional selves. They also describe locum doctors feeling stuck in permanent liminality. Similarly, Croft et al. (2015a) describe nurses moving from clinical into ‘hybrid’ managerial roles experiencing a ‘*perverse* liminal space’, in which many felt unable to be either nurse or manager, that undermined identity transition, resulting in nurses leaving the health workforce. Conversely, Chen and Reay (2021) describe occupational health professionals using liminal role transitions to reconstruct positive new professional identities. Thus, the very limited number of studies of health workers’ experiences of liminality show it having (often negative) implications for their practice, identities and careers.

### 2.2 Liminality in health care in LMICs

In LMICs, due to severe resource constraints, health professionals and public servants often have to enact ‘practical norms’, which refer to informal norms that underlie practice but deviate from official or professional norms (Herdt and Sardan, 2015). These may be ‘palliative practical norms’ required to maintain provision of public service where resources to meet official standards are unavailable. However, the same conditions may also enable ‘transgressive practical norms’, such as corruption, nepotism, negligence and malpractice (Herdt and Sardan, 2015; Nzinga et al., 2019). These studies suggest that, in the context of severe resource constraints, professional practice is inherently liminal as it is situated between official, professional, practical, palliative and transgressive norms.

In Kenya, as in other LMICs, health professionals are usually trained in large, relatively well-resourced university-affiliated hospitals, where trainees have little exposure to the practical norms of resource-constrained clinical practice. After undergraduate training, medical and nurse ‘interns’ are posted to internship centres that are relatively poorly resourced with equipment, medications, diagnostic equipment, and staff (Zhao et al., 2022). Thus, during internships these health professionals are often unable to practice in line with professional standards and norms inculcated during undergraduate training, and so have to learn the practical norms of professional practice (Mbuthia et al., 2021; Muthaura et al., 2015). In this paper, in our first ‘practice’ dimension of liminality, we were interested in understanding how new professionals navigate experiences of liminality associated with transitioning from undergraduate training into professional practice, and how they learn to enact new practical norms in their day-to-day practice.

New doctors and nurses also need to learn how to relate to members of other health professions. Stein (1967) described a liminal and relational ‘doctor-nurse game’ in which doctors demonstrate their superior expertise and status over nurses, while relying on nurses to perform clinical work, and nurses accept doctors’ superiority, whilst indirectly influencing them. Stein (1967) suggested that while nurses learn this liminal ‘game’ during training, doctors do so during internships.

Forging connections across professions is an important aspect of healthcare delivery. For new health professionals, positive inter-professional relationships can build legitimacy in their career choice and professional identity (Chen et al., 2022). However, we know little about how new professionals experience and learn to negotiate these liminal inter-professional boundaries and relationships, particularly in resource-constrained LMIC settings. Research in the Kenyan health system suggests that unclear professional hierarchies, relations and pervasive mistrust pose a challenge to nurturing positive relationships (Nzinga et al., 2021). Under our second ‘relational identity’ dimension, we investigate how new health workers negotiate the liminal boundaries and relationships between professions with competing notions of acceptable practical norms, experience, expertise, and status.

In complex and changing professional contexts, there may be variations *within* professions regarding perceptions of appropriate practices, roles, values, behaviours or (mis)conduct (Anteby et al., 2016; Boateng and Adams, 2016; Jepkosgei et al., 2022; McGivern et al., 2015). There may also be competing professional subspecialties (Croft et al., 2015a; McGivern et al., 2015; Zetka, 2001). Relational inconsistency, lack of support and influence in group membership within relational spaces may threaten professional identities, strain new health professionals’ emotional and psychological well-being, leading individuals to separation from the professional group (Croft et al., 2015b). Studies suggest that managers in some resource-constrained contexts find it difficult to identify with the emotional state of the collective (Nzinga et al., 2021). Within our second dimension of liminal relational identity, we therefore also examine how liminal experiences of *intra-*professional differences operate and affect the dynamics of care delivery at the micro-level (Boateng and Adams, 2016).

Indeed, professional identities are constructed within intra- and inter-professional relational spaces (Kreiner et al., 2006). Relational interactions with members of one’s own and other professions may surface professional boundaries, hierarchies, differences, competition, and conflict between and within professions, which are maintained and transformed through the everyday ‘identity work’ of professionals in practice settings (Croft et al., 2015b; Ibarra, 1999; Kreiner et al., 2006; Mbuthia et al., 2021; McGivern et al., 2015; Nzinga et al., 2019; Pratt et al., 2006; Waring et al., 2023). We explore how new health professionals in our studies negotiate liminal experience of differences within and between professions professional while reconstructing their professional identities.

Lastly, in relation to our third dimension of liminal careers, the literature suggests that most doctors and nurses undertake clinical training expecting permanent, prestigious and lucrative careers (Gąsiorowski et al., 2015; Mbuthia et al., 2021). Yet many find themselves in temporary and precarious professional employment, with limited avenues for professional advancement (Maleki et al., 2021; Zhao et al., 2023c). This echoes the notion of ‘permanent liminality’ or ‘limbo’ (Johnsen and Sørensen, 2015; Söderlund and Borg, 2018; Ybema et al., 2011), ‘occupational limbo’ (Bamber et al., 2017) and ‘perversive liminality’ (Croft et al., 2015a) described above.

In Kenya, following the recent devolution of government functions, authority has shifted from national to subnational levels, contributing to the transformation of the employment landscape. While the number of medical and nursing schools has expanded, the recruitment of newly graduated doctors and nurses into the public sector has not increased at the same pace (Okoroafor et al., 2022; Tsofa et al., 2017; Zhao et al., 2023c). Thus, Kenyan interns may experience uncertainty about their future professional careers. We investigate how health professionals respond to unexpected uncertainty and liminality relating to their career advancement.

In sum, we explore how new health workers experience liminality in relation to their professional practice, relational inter and intra-professional identity and status, and careers in the face of severe resource constraints in Kenya.

## 3 Methods

This paper is based on three sub-studies examining Kenyan doctors’ and nurses’ experiences of transitioning from University (medical and nursing school) into clinical practice. These sub-studies formed part of a larger project examining human resources for health management in Kenya. Ethical approvals for the study were issued by [Kenya Medical Research Institute (KEMRI) (SERU 4071)] and the ethics committees in the authors’ institutions. Informed consent was obtained from all interview participants.

Sub-study A examined nurses’ transition from undergraduate training into clinical practice during one-year internships in Kenyan hospitals. It drew on 55 semi-structured individual interviews and seven focus groups, involving 30 intern nurses, interviewed at the beginning and end of internships. Sub-study B examined medical students’ expectations and experiences of one-year internships following graduation from medical school, drawing on 18 semi-structured interviews with final-year medical students and 33 medical interns. Sub-study C examined recently qualified doctors’ internship experiences and career transitions, drawing on interviews with 30 medical officers (junior doctors) who recently completed internship, and ten senior doctors involved in internship supervision. In total, then, the paper draws on 146 individual interviews and seven focus groups involving 121 participants. All three studies were conducted in the same period, during the Covid-19 pandemic, resulting in many interviews being conducted online through Zoom or Microsoft Teams. We show details of the three sub-studies in Table 1.

Our project involved an ‘insider-outsider’ research team drawing on different perspectives (Louis and Bartunek, 1992; Thomas et al., 2000). Three authors are Kenyan nationals living in Kenya, two are British citizens who lived and worked in Kenya, and three have conducted empirical research in Kenya but have not lived in the country. One of the authors practised as a doctor and one as a nurse in Kenya. The other authors have public health and social science training. While data collection for the sub-studies were conducted by different team members, the wider study team met monthly for over two years to discuss findings, with their different experiences and perspectives helping analysis and interpretation of research findings.

**Fig. 1:**
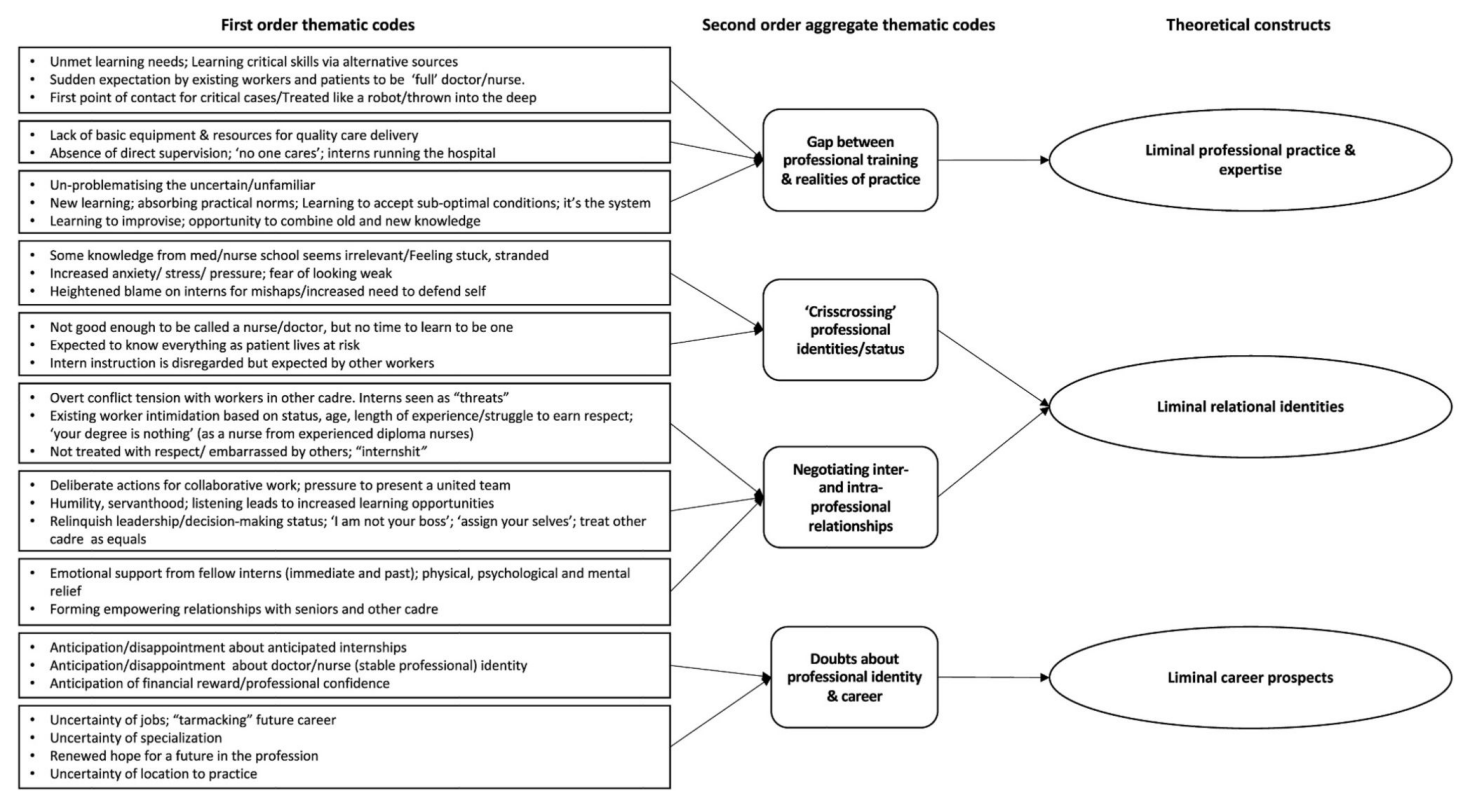
Data structure.

We coded and theorised our qualitative data using an inductive and iterative thematic approach (Gioia et al., 2013). Discussing common themes across sub-studies led us to consider ‘liminality’ as a theoretical frame explaining common findings in relation to experiences of internships. The authors shared literature on liminality and participated in six analysis meetings during which we discussed theoretical literature and empirical data. We identified the three common ‘liminal’ themes discussed above. To test and apply these themes rigorously across the sub-study datasets, each team then organised their coded data into matrices. We discussed findings in subsequent meetings, prompting further examination of sub-study data. Thus, we iteratively moved between empirical data and theory (Gioia et al., 2013), as illustrated in Fig. 1.

## 4 Findings

We now discuss our empirical findings structured around the three themes of liminality of (1) liminal professional practice and expertise; (2) liminal relational identities, and (3) liminal career prospects and realities (Fig. 1). These interlinked themes affected new health professionals ongoing career decisions and professional identity formation.

### 4.1 Liminal professional practice and expertise

Most interns we interviewed described being unable to apply knowledge learned during their formal undergraduate medical and nursing training or practise as the health professionals they expected to be during internships. Interns undertook undergraduate training in relatively large, well-equipped, university-affiliated hospitals, and then did internship training in smaller, less well-resourced hospitals. This transition created confusion for intern doctors and nurses about how to meet practice standards and use knowledge inculcated during undergraduate training.

#### 4.1.1 The gap between professional training and practice

Interns discovered they were the first point of contact for patients. Many felt *‘thrown into the deep end’*, especially when supervision was unavailable. Lack of supervision also affected learning, with interviewees expressing frustration on discovering they were often running wards and that senior colleagues did not seem to care about patient quality of care. Without guidance from seniors, interns felt that they were expected to practice as fully trained professionals, despite having limited experience. To cope without supervision, some interns had to either use learning sources such as Google or *“YouTube”* (Medical doctor 4), or make do with their own limited knowledge, sometimes with negative implications:

“If [interns] had someone who will show him [how to do] the first Caesarean Sections (CS) and say, ‘Okay, cut here, do it like this, remove it like this”, once you do it, the next time, it will be easier. But [in internship] you are left alone, you go and operate on a patient, you make an incision until you cut intestines, because you don’t know how much pressure to put.” (Medical student 02).

Most doctors agreed that, without supervision, on-the-job and improvised learning was necessary. During formal training, interns had been trained to make clinical judgements and decisions based on evidence. Yet during internships, without the resources, equipment and diagnostics they needed to carry out investigations in line with formal training, interns were *“forced to treat and manage patients purely on the clinical acumen, and not [based] on evidence-based medicine”* (Medical doctor 11). Interns also had to work long hours, manage high numbers of patients. Thus, as one doctor described, *“being in hospitals where resources are constrained, you realise that there is some work you would want to do but you can’t do them in the appropriate time.” (Medical doctor 15*).

Similarly, most nurse interns observed a gap between what they were taught in nursing school and observed in practice. They described senior staff breaching nursing practice standards, for example, writing notes in advance of giving care, combining antibiotics, and giving drugs before scheduled timings. Some nurse interns even described colleagues giving high doses of oxytocin to expectant mothers to induce labour, contrary to doctors’ prescription, so that mothers gave birth quickly and nurses could go home, or sleep on night duty.

“When I was in the wards doing my medical rotation, I was really frustrated a lot of the time because of the quality of care that was being given, because of the disparity between what is supposed to be done and what is being done, and how nobody cares.” (Nurse intern - FGD2)

Due to resource constraints and being exposed to ‘transgressive practical norms’ (Herdt and Sardan, 2015) and practices described as “*medical witchcraft*” *(Nurse intern - FGD 2)* which were dangerous to patients, interns faced professional dilemmas. For example, a nurse intern described her sense of confusion when she discovered that the hospital had no oxygen and nasal prongs (tubes which oxygen is delivered through into the nose) for newborn babies. Instead, the hospital used nasogastric tubes to administer oxygen to patients.

Interns struggled to adjust to practical norms deviating from official and professional practices learned during undergraduate training. Their concerns were exacerbated by the high numbers of patient they were expected to care for, often alone. For example, Nurse intern 25 commented: “*When I came [into internship], I was left with an entire ward on my own, I didn’t even know how to manage it”*. Being left alone to make critical decisions, affected some interns psychological and emotional wellbeing, resulting in “miserable” and “inhumane” *(Medical intern 02)* internship experiences. Medical intern 18, described how he *“almost lost his mind”* after being pressured to care for over 100 patients, and sought counselling as he felt no one in the hospital understood the pressure he experienced.

However, other interns came to understand that sub-optimal professional practices were required to maintain health care in poorly resourced internship hospitals. In theoretical terms these interns considered these practices as ‘palliative practical norms’ (Herdt and Sardan, 2015). This understanding provided interns some comfort that they were meeting their duty of care. Indeed, gradually, interns generally learned and came to accept sub-optimal clinical practice, palliative practical norms, shortcuts, and workarounds as necessary in internship centres without the ‘ideal setup’ of training. As one nurse described:

“There is a way that one [oxygen] cylinder can be divided for three or four babies. I didn’t learn that in nursing school but that is how you can ensure that three or four babies get that oxygen delivered.” (Nurse intern - FGD 2)

Thus, interns learned, absorbed, accepted, and normalised liminal practices they had initially been shocked by, creatively improvising sub-optimal practices using protocols and limited resource.

### 4.2 Liminal relational identity

Our data suggests that liminality was constructed in relation to professional groups and status.

#### 4.2.1 ‘Criss-crossing’ professional identities and status

Many interns expressed concern around enacting their liminal responsibility, professional identity and status in relation to inter and intra-professional dynamics. For instance, one medical intern described the *“confusing”* and *“terrifying”* experience of suddenly moving from being a medical student to being a real doctor: *“You are taught about the decisions [as a medical student], but you never really make the decisions. Then, one morning, you wake up and it is like: ‘Daktari (doctor), what do we do? ‘Actually, I was terrified*” *(Medical intern 20)*.

Some experienced confusion about being expected to be patients’ first point of contact whilst simultaneously being belittled, treated like students, and having their opinions about patient care disregarded my more experienced colleagues. One Intern doctor described this as being *“in between a student and being a medical doctor … but not good enough to treat patients” (Medical doctor 09)*. Other medical interns felt that their perceived lack of clinical credibility deprived them of meaningful experiences and threatened their professional identity, especially when senior doctors kept them away from more challenging tasks: “C*an you imagine working with your senior, who never even allows you to take part in certain procedures, just because they feel like you will not do a good job?” (Medical doctor 1*).

Nurse interns, trained to graduate level, described how their formal knowledge ‘threatened’ experienced nurses trained to diploma level: *“Leadership does not really appreciate BScN. Because, firstly, they see us as threats. We have more knowledge or theory. It doesn’t make us colleagues; it makes us more of a threat.” (Nurse intern - FDG 2)*. This contributed towards nurse interns experiencing a sense of liminal uncertainty about how to relate other nurses in the clinical environment.

Nurse graduate interns also described senior diploma-level nurses depriving them of opportunities to apply their learning during undergraduate training. For instance, senior nurses would not allow graduate nurse interns to perform procedures they were trained to do, like inserting catheters, nasogastric tubes and intravenous lines, as they believed these procedures should only be conducted by doctors or clinical officers. When interns asked to perform these procedures, they were called “know-it-alls”, which they experienced as demoralising:

“A nurse wouldn’t catheterize a patient … [or] put in a NG (nasogastric) tube because it’s not a nursing role … When you want to do these things, they [experienced diploma-level nurses] start saying, “you know-it-all, let the CO [clinical officer] come and do that”. But now what am I supposed to be doing as a nurse? What do people do here as a nurse? They only give medication, receive the Cardex and your work is done. You can sit and wait for your shift to end and go home!” (Nurse intern - FGD 2)

Intern nurses’ inability to immerse themselves in the nursing role and their professional group led to them being to be blamed for mistakes too. A nurse intern stated:

“You have to make sure they [students] don’t make mistakes, because you will be blamed for them. Your name is always there on the radar for any mistake, you are held accountable.” (Nurse intern 02)

Some medical and nursing interns described feeling like the ‘most harassed’ workers in the hospital environment.

Interns struggled with a sense of liminality tension as they learned how to navigate complexity and uncertain in relationships and related levels of expected expertise within and between health professions. Interns described how more experienced health workers paradoxically expected interns to perform critical tasks competently without support, while simultaneously viewing interns as incompetent. For instance, new medical interns described how nurses refused to support interns due to interns’ superior status and pay as doctors, as an intern doctor described:

“Nurses sometimes don’t give you that support that you need. They just tell you: “you are the doctor … You are being paid a lot of money … You need to know how to do this.” (Medical doctor 3).

Indeed, many interns described “*struggling to earn the respect”* of more experienced workers in other professional disciplines. Intern doctors commented on being belittled and intimidated by nurses due to their intern status, young age, and limited experience. For example, a medical intern noted: *“a number of [nurses] are very mean; they will treat you like you are nothing and they will tell you that there is nothing you can do” (Medical intern 32)*.

Other interns were caught in team conflicts they knew nothing about and as interns could not resolve, as another intern doctor described: *“Some [nurses] have been working here for years. When one is insisting on something and another one is insisting on other things, that becomes a big problem. They look at you like: ‘what can you tell us, you just keep quiet and let us deal with this” (Medical intern 08)*. This was difficult for medical interns who, as doctors, were expected by senior colleagues to lead teams and present a “united front” during patient case presentations while, as medical intern 08 commented, *“in your head, you know, you are not a team anymore*.”

Thus, inter- and intra-professional relations created a liminal state in which interns were constrained in enacting their desired professional identity and integrating into their profession, while at the same time being unable to enact their former student identities.

#### 4.2.2 Negotiating inter- and intra-professional relationships

Learning to negotiate liminality in relation to inter- and intra-professional relationships was a key aspect of the internship experience. Making official complaints about bad behaviour was interpreted as being *“weak” (Medical doctor 02)*, so instead interns had to learn to influence health workers.

Medical interns, as medical professionals, were higher in the informal professional social hierarchy than diploma-level nurses. However, medical interns were unable to use their status as medical professional to influence nurses. Instead, medical interns learned to influence nurses by transcending different levels of professions’ status and expertise, “going down to their level” and treating nurses “as equals”:

“If you come to them and show them that you are a doctor, then they won’t be nice to you. I go down to their level and talk to them, as if I am the servant, they are my master, and when you do that to them, life becomes so good.” (Medical intern 05)

“Nurses can really mess you up … but once you approach people as equals, they are able to get to your level. Some of them have been in the profession for, like, thirty years … You are seeing it for the first year.” (Medical intern 15).

These relational tactics provided interns some agency and influence during clinical practice.

Nurse graduate interns describe how they learned to influence less qualified colleagues to change their practices by politely using knowledge of research and official clinical protocols: *“Convince them in a good way like: ‘You know Sister, while you do this and this, research says this and that, and the current protocol says this’. Most of them will buy your idea” (Nursing intern 28)*. Thus, through humility, treating others as equals, and drawing attention to protocols, interns learned to influence other professionals and clinical practice.

Many interns received support from more experienced interns, who provided encouragement, advice, and shared stories of past similar experiences and overcoming emotional difficulties, which provided important psychological support to new interns:

“Just calling up my colleagues, the interns that were ahead, asking them: “how did you survive?” Getting stories, so that if something happens and I don’t know how to manage, I know, okay, someone told me they did this, then I do that exact thing.” (Nurse Intern - FGD 1).

Thus, supportive intra-professional relationships with more experienced interns helped new interns make sense of their liminal experiences and reconstruct their professional identities:

“The only support I think I got was from my fellow interns. We encourage each other along, because at times things may get quite tough … They are the ones that give you the emotional support.” (Medical doctor 18)

Indeed, many interns formed informal networks and groups that shared practical experiences, which enabled them to cope with liminal clinical practices, relational identities and statuses within professional hierarchies.

### 4.3 Realising the reality of liminal careers

Most interns we interviewed had aspired to join a health profession all their lives and expected rewarding careers after internship. However, although internship provided temporary employment, interns were concerned about their uncertain future careers. The severely resource-constrained Kenyan health system and the changes in its employment landscape perpetuated feeling of permanent future liminality. One medical intern (28) commented “*getting employed is hard … There is no assurance of a permanent posting. We are really scared about it [post internship] because there is no employment.”* Other interns described feeling that they would be *“thrown under the bus immediately after internship” (Medical doctor 27)* due to disappointing career options. Similarly, a nurse intern described feeling like a “ghost”, abandoned, and forgotten immediately after internship, with few job opportunities.

“If an opportunity comes my way, I will just grab it. Irrespective of the pay, I will still apply, because you don’t see [graduate nursing job] posts anywhere … We become ghosts after internship. There is nowhere that we are mentioned even in [job] posts.” (Nurse Intern 01)

Interns described limited prospects for advancement and professional development after internships, and therefore feeling able to achieve their expectations of the professional identity. One nursing intern (09) stated *“I feel like I am doing things that are not in the scope [of professional practice]. I do not see growth where I work.*”

#### 4.3.1 Doubts about professional identity and career

Some interns used the expression of ‘tarmacking’ to describe an experience of being in a liminal career state, struggling to find a job, with the possibility of an irrecoverably disappointing future professional identity, as illustrated by a medical student:

“There was this talk that when you do medicine … your future is sure because there are a lot of opportunities that come from it. You will almost hardly miss an opportunity to be gainfully employed after finishing University. But now, we’re being told people are tarmacking.” (Medical student 01)

The metaphor of ‘tarmac’ describes internships and subsequent health professional careers as an initially malleable and fluid liminal state in which professionals mould their professional identity to fit any available clinical roles before settling into a stable state. Some interns, such as those with large families that depended on them, described needing to do any clinical work, even when stuck in unsatisfying temporary clinical roles, so that “at least you can put food on the table” *(Medical intern 24)*. However, this stable state may then leave new health professionals stuck in limbo, without opportunities for career advancement or fulfilling their professional identity and status aspirations.

Many intern interviewees described needing a “Plan B”, “to find a way out” of this professional career limbo. For example, by continuing in formal education, taking a Master’s degree program, although they also feared returning to occupational limbo state. Others hoped to escape the tarmacking state by taking jobs outside health care or Kenya. For example, one medical intern described taking up fish farming to save up for a clinical Master’s degree. Others planned to leave Kenya and work in better-resourced countries, where health workers are in more demand. We discuss the liminal metaphor of ‘tarmacking’ further below.

## 5 Discussion

We examined the experiences of 121 new doctors and nurses transitioning through internships from medical and nursing school into clinical practice in the highly resource-constrained Kenyan public healthcare system. We used ‘liminality’ as our conceptual frame, which has been overlooked in studies of new health professionals’ career and role transitions, particularly in resource-constrained contexts.

The internships we examined were structured and collective liminal processes, with a clearly defined rite of passage from one stage (pre-service education) to the next (fully qualified health professional). Internship training is highly institutionalised but complex, with different cohorts of interns starting and ending at the same time.

Our analysis of interns’ transitional experiences highlights three forms of liminality relating to professional practice, relational inter- and intra-professional identity and status, and professional careers. By explaining these three distinct aspects of liminality together, we provide a novel conceptual framework for understanding health workers’ liminal experiences of career and role transition. Our study also extends the discussion of liminality to health professionals in highly resource-constrained contexts.

### 5.1 Liminal professional practices

Our first contribution is to characterise the liminal nature of professional practice and how it is learned in resource-constrained health systems. Medical and nurse interns are expected to deliver frontline patient care as soon as they begin their internships (Ogero et al., 2020). However, during internships these new doctors and nurses discovered that many of the best practices they learned during medical and nursing school cannot be enacted in resource-constrained public hospitals. Thus interns learned ‘practical norms’ (Herdt and Sardan, 2015) and related practices to maintain provision of health care, often without supervision from senior colleagues, and sometimes using YouTube and Google for guidance. While research (Mbuthia et al., 2021; McKnight et al., 2020) has discussed the challenges of professional practice in resource-constrained settings, sometimes involving using ‘practical norms’ (Nzinga et al., 2019) or ‘improvising medicine’ (Livingston, 2012), less focus has been paid to its liminal nature. By analysing these resource-constrained using the concept of liminality, we provide new insights into health workers’ experiences and their implications for health care.

New doctors’ and nurses’ experiences of transitioning through internships were characterised by liminal practice in several ways. These experiences involved abandoning expectations of providing high quality care and embracing previously unimaginable practical norms and professional practices (‘medical witchcraft’, ‘inhumane’ practices) as ways of coping. Such practices and ‘workarounds’ (Amalberti and Vincent, 2020) are also liminal in that they are professionally, ethically and legally ambiguous (Zhang et al., 2023). ‘Transgressive practical norms’, such as malpractice, negligence or corruption, clearly compromise patient care and interns’ professional identities. However, ‘palliative practical norms’ (Herdt and Sardan, 2015), such as improvising with limited equipment and resources to maintain the provision of patient care in resource-constrained conditions can be considered ethical good professional practice (Debono et al., 2013; Herdt and Sardan, 2015; Mbuthia et al., 2021; Nzinga et al., 2019).

Internships involved distinguishing between necessary palliative practical norms and transgressive practical norms and related sub-standard professional practices harmful to patient care. However, interns were left alone to navigate, interpret and make ethical judgements and decisions about such norms and practice, which many found emotionally demanding, which lead to moral distress (Nzekwu et al., n. d.). Such liminal practices are likely to become increasingly common in health care and may remain liminal even as health professionals become more experienced, given the growing resource constraints in health care globally. Under these conditions, professionals may therefore experience perpetual liminality clinical practice.

A key policy recommendation from this finding is that training needs to better prepare new health workers for liminality they will experience when beginning clinical practice, which is often very different from best practices currently taught during undergraduate level. For example, the nursing preceptorship socialisation program in high-income countries has been helpful in navigating liminal practice and raising awareness of ‘real’ nursing practice (Billay et al., 2015). Furthermore, working as a new nurse or doctor, particularly in resource-constrained contexts, requires clinical and ethical judgement. Thus, health workers need to be better prepared to improvise care and enact professional ‘ethics on ground’ where best practice is sometimes impossible (Heimer, 2013).

### 5.2 Liminal relational identity

Second, we contribute to the literature on liminality by drawing attention to health professionals’ liminal relational identities and status. Inter- and intra-professional relationships are widely discussed in the professions and healthcare literature (Anteby et al., 2016; Jepkosgei et al., 2022) and are crucial for health care decision-making and delivery. However, the liminality that new health professionals experience as they learn to relate to members of their own profession, and other professions, they work has been overlooked in existing health professional literature, particularly in resource-constrained contexts.

Our study offers new insights into how the development of new health workers’ professional identities can be intentionally or unintentionally undermined (or supported) by senior or experienced members of their own and other professional groups. Our study shows how relations between collective professional groups create liminal relational space that new health professionals must learn to negotiate to develop their professional identities and practices. Thus, we show how liminal professional identities and statuses are relationally imposed and shaped.

In their study of leader identity construction, Croft et al. (2015b) show the significance of acceptance by the collective profession in constructing the desired professional identity. Our study builds on this by highlighting the way that new health workers negotiate and sustain a liminal connection with their desired professional identity and status.

Interns in our study negotiated challenging relationships with members of other professionals, by ‘being humble’, dropping aspects of their professional identity (for example, as medics being high status and ‘in charge’), and ‘coming down’ to status level of other health professionals they were working with. Although their descriptions suggest they could have been better prepared for these unexpected encounters, their response was constructive in accomplishing a dual function: maintaining the pursuit of their desired professional identity and acceptance by the collective profession. Although embracing a lower identity and status may be conceived as weak, it could also be seen as skilful relational work in the face of emotionally and practically challenging inter and intra-professional relations.

Our study provides insight into new health workers’ *emotional* struggles with clinical practice in severely resource constrained health care contexts. Existing studies highlight challenges faced by many health systems in resource-constrained settings, which create fertile grounds for distrust making positive behavioural and relational interventions unsustainable (Nzinga et al., 2021). Interns in our study described supervisors behaving in unprofessional or unsafe ways, being unsupportive and abusive, and blaming interns for failures resulting from wider problems in health systems.

Prior scholarship shows that the lack of support from professional groups can perpetuate negative emotional experiences (Croft et al., 2015b). Yet, while for some this may become overwhelming, our study suggested that an emotionally supportive intra-professional groups and networks, involving interns from the same training cohort or who had recently completed internship, helped navigate this. Meeting in ‘psychologically safe’ (Edmondson, 2023) spaces helped interns support each other emotionally, practically, and to interpret and survive the liminal experiences of internship. Other research examining Swartz Rounds (Lown and Manning, 2010) and Balint Groups (Kjeldmand and Holmström, 2008) has also highlighted the key role that such spaces have for health professionals. By highlighting this process for surviving liminal identity and status, our study adds to research on new health professionals’ emotional experiences during identity construction.

### 5.3 Liminal career prospects and realities

Our third theoretical contribution highlighting the realities of liminal health professional careers in resource constrained contexts, reflecting previous studies describing permanent liminality or occupational ‘limbo’ (Bamber et al., 2017; Garsten, 1999; Johnsen and Sørensen, 2015; Söderlund and Borg, 2018; Ybema et al., 2011). The healthcare literature has also discussed liminality among temporary workers, like locum doctors (Gordon et al., 2020) and ‘gig economy’ nurses (Lien, 2023), and nurses experiencing ‘perverse liminality’ in ‘hybrid’ clinical-managerial roles (Croft et al., 2015a).

Internship necessarily involves liminality, as it is a transition from one stage (medical and nursing school) to another (clinical practice). However, this is also dependent on the external environment. New doctors and nurses in our study discussed limited future job, training and career opportunities in the Kenya health system (also see Zhao et al. (2023c, 2023a). Reflecting on broader literature on liminal work (Johnsen and Sørensen, 2015; Obodaru and Ibarra, 2016; Ybema et al., 2011), the new doctors and nurses we interviewed described feeling ‘stuck’ while seeking alternative careers, conveying a sense of permanent liminality continuing after internships into their professional careers.

We draw attention to the metaphor of ‘tarmacking’ that interns described in relation to their liminal career prospects. Tarmac, as a substance, is temperature dependent, moving from fluid and ‘sticky’ when hot to solid (and facilitating movement) when cool. The tarmacking metaphor may explain how health workers cope with heated (emotional) ‘sticky’ liminal career phases, while ‘cooly’ (rationally) maintaining hopes for desired durable professional identities and status. However, the tarmacking metaphor also communicates a sense of being stuck in ‘perverse liminality’ (Croft et al., 2015a), permanent liminality in temporary roles (Johnsen and Sørensen, 2015; Söderlund and Borg, 2018; Ybema et al., 2011) or ‘occupational limbo’ unable to achieve a desired professional identities or status desire (Bamber et al., 2017). However, through skilful improvisation the interns we interviewed also described constructing ‘new avenues’ towards future careers that enabled them to escape the stickiness of tarmacking.

Liminal careers are increasingly common for health professionals (Gordon et al., 2020; Lien, 2023), causing widespread changes to professional identities and expectations. Studies also show the fluidity of health workers careers along international migration pathways (Bourgeault et al., 2023). For example, an increasing number of doctors and nurses in the UK have moved to work in Australia and New Zealand for similar reasons found in our data - the lack of quality healthcare jobs (Brennan et al., 2023; Smith et al., 2018), making post-internship extremely unpredictable.

In LMICs where there are severe workforce shortages, migration of health workers to high-income countries immediately after qualification as a result of unemployment and underemployment is a major system failure. Our study highlights the necessity of policies for workforce planning, recruitment and retention specifically for intern health workers as part of building wider health system resilience (Witter et al., 2023).

Finally, our study is subject to limitations which provide opportunities for future related research. While concept of liminality provides a useful theoretical framework for explaining our data, we did not explicitly explore this concept in interviews. Furthermore, our study of new doctors and nurses was conducted in a single country (Kenya) and during the Covid-19 pandemic, which provided a particular context that both limited our data collection (many interviews were conducted online) and impacted interns’ learning and transition experiences and broader labour market dynamic. Therefore, future research explicitly testing our framework with interviews with other health professionals, including other interns, going through role and careers transitions are needed in other policy, organizational and national contexts.

## 6 Conclusion

We examined medical and nurse interns’ liminal experiences and related processes of professional identity reconstruction as they transitioned through internship training in the resource constrained Kenyan health system. We outline a conceptually novel framework showing three liminal experiences that new health professionals experience. First, the liminal nature of their professional practice as they encounter practical norms and realities of working in a resource constrained health system. Second, liminality in relation to initially learning and then continuing to negotiate *intra*-professional relationships with supervisors and peers and *inter*-professional relationships with other health professionals, and how new health workers relationally construct their professional identities and status. Third, coming to terms with and responding to the reality of liminal career prospects after internship. Our findings resonate with existing literature on liminality, permanent liminality and occupational limbo, while extending their analysis into resource-constrained and LMICs contexts, and explain how new professionals learn, respond to and are affected by liminal inter- and intra-professional relationships.

We call for further studies with a specific liminality lens to explore this critical period in health workers’ careers. A deeper understanding is essential to inform policy and practice that better responds to the changing nature of heath care practice and health professions in Kenya, other LMICs and globally.

## Supplementary Material

Supplementary Material

## Figures and Tables

**Table 1:** Data sources and collection.

	Sub-study A: Graduate nurses’ transition from training into clinical practice during internship	Sub-study B: Medical students’ expectations and experiences of clinical practice during internships	Sub-study C: Medical officers’ reflection of internship experiences and subsequent career transitions
Study population	30 Bachelor of Science Nurse Interns	18 final year medical students 33 medical officer interns	30 medical officers (within 3 years of completing internship) 10 consultants
Respondent characteristics	All from University X Posted across 20 county hospitals (urban and rural settings)	All final year medical students from University X All medical officer interns within the first 12 months of entry into internship hospitals (public hospitals across 11 counties and 1 private hospital) who received their undergraduate training from University X	30 medical officers from 6 different undergraduate universities and in 25 different internship hospitals, currently mostly working in clinical practices and 3 work in research or business 10 consultants across four different specialties in 8 different hospitals
Sampling	Convenience sampling approach; Participation was voluntary, initial engagement was with the graduates’ class of 40 participants. Those interested were asked to contact the study team via email	Snowballing approach through facilitated introductions or referral by an interviewee; interns working in different hospitals	Snowballing approach through facilitated introductions or referral by an interviewee; individuals were selected also considering different internship hospitals, current occupation and for consultants’ different specialties
Data collection approach	Focus group discussions and semi-structured individual interviews lasting 45 min to 2 h Online via Microsoft Teams between Feb 2021 and Feb 2022	Semi-structured interviews lasting 60–90 min Online (Zoom) in English between March and August 2021	Semi-structured interviews lasting 20–60 min In-person or online (Zoom) in English between June and Sept 2021
Data collection focus	Preparedness for internship	Expectation of internship Preparedness for internship Internship experiences	Wellbeing, educational and work environment during the internship Experiences of applying and securing post-internship jobs

## Data Availability

The data that has been used is confidential.
