# Supplementary material for: Examining liminality in professional practice, relational identities, and career prospects in resource-constrained health systems: Findings from an empirical study of medical and nurse interns in Kenya
Source: Soc Sci Med. Author manuscript; Available in PMC 2025 Jun 21. (PMC7617787; doi:10.1016/j.socscimed.2024.117226)
Supplement: Supplementary Material [file EMS206504-supplement-Supplementary_Material.zip › 1-s2.0-S0277953624006798-mmc1.docx]

**Supplementary appendix 1. Interview and focus group guide**

# Sub-study A: Graduate nurses’ transition from training into clinical practice during internship

***Interview with trainee nurses pre-internship***

1. What were your reasons for studying nursing?
2. How was nursing school for you?
3. Did you find any professors particularly inspiring nursing school? What did they do that inspired you? What about the senior students influence?
4. How do you see your nursing career ahead?
5. What do you expect your internship to be like? **Probe** What are you most looking forward to?
6. How prepared do you feel for nursing practice? **Probe**: Do you have any worries about internship?
7. What aspect of your training do you feel best prepared you for nursing practice? (**probe** on technical/communication aspects/management of emotions).
8. How were your clinical placements at KNH? How was the support from the nurses like? How was the support from the clinical instructors?
9. What aspect of practice do you think would be more challenging**? Probe:** Are there any things you feel unprepared for?
10. Do you think your peers would face similar challenges or is this personal to you?
11. What can you say about the role of NCK as a body of Nursing in Kenya? What have they done well? Are there any areas that you think they need to improve? What can you say about the scope of practice for BscN?

***Focus group with trainee nurses pre-internship***

1. What are the main strengths of the nursing school in preparing students for nursing practice? What are some of the main challenges? Are there any groups of students who face more challenges? (e.g background, gender, where from etc).

Probe: Not all students initially choose a nursing career but find themselves in nursing school. Do these students face any struggles? How can such students be identified and how can they be supported?

1. What aspect of nursing practice do you feel nursing students are best prepared for before their internship? And least prepared for? Prob Technical ability? Ability to communicate with patients/colleagues/managers? Handling of emotions?
2. How well do you think students are prepared for their internships? In what areas would you say they are best prepared for and why? In what areas are they least prepared for and why?

Probe: Are there any areas in the clinical placement that needs to be improved to prepare students better for their internship and (and future careers?) in your view? How might this be done?

Probe: How do placements at KNH help prepare students for their internship and nursing practice? (opportunities, challenges?) How well are students supported in these placements? How do BScN students relate with KNH nurses during these placements?

1. What are students’ expectations for the internship and does the experience so far meets their expectations? What are some of the worries that students have about the internship?
2. In your view, where do most BScN interns wish to work and why? What informs these choices?

***Interview with trainee nurses end-of-internship***

1. How was the internship?
2. Could you please tell me about any experiences and events which were significant for you during your internship (start wherever you like)?
3. How well prepared do you feel you were for nursing practice during the internship?
4. Which aspects of nursing practice did you feel particularly well or poorly prepared for? Can you describe any examples of this? (probe on technical/ any experiences/ challenges on communication with patients /management of emotions)
5. What were the biggest unanticipated challenges you faced during the internship? Probe - what can you say about the support you received at the hospital as an intern?
6. How did you deal with these challenges?
7. How has your experience of the internship affected your views of nursing and clinical practice?
8. Were there any people who played a particularly significant (positive or negative) role during your internship?
9. Do you have any suggestions for better preparing nursing trainees for their internship? If so, what would it be?
10. How has the internship affected the way you see your nursing career?
11. What are your plans moving forward?

***Focus group with trainee nurses end-of-internship***

1. What are interns main expectations of the internship ? And in what ways were these expectations met?

Probe: What are the biggest unanticipated challenges that interns face during their internship? Probe: In what ways do interns deal with these challenges?

1. How well do you think interns are prepared for nursing practice ? In what areas would you say they are best prepared for and why? In what areas are they least prepared for and why? Prob Technical ability? Ability to communicate with patients/colleagues/managers? Handling of emotions?

Probe: what can you say about the support interns receive at the hospitals to support their transition?

Probe : Are there any people who play a particularly significant (positive or negative)role during the internship.

1. In what ways does the internship experience affect interns views of the nursing career and clinical practice? Probe: Are there any areas that needs to be improved to prepare interns better for internship (and future careers?) in your view? How might this be done?
2. In your view, where do most interns wish to work after the internship and why? What informs these choices?

# Sub-study B: Medical students’ expectations and experiences of clinical practice during internships

**Questions to final year medical students**

Goal: to understand their current expectation and the anticipated experience of clinical practice during internship.

1. **Opening/General question**

- Tell me about what you did while waiting to begin your internship.
- What personally attracted you to the medical profession?

1. **Trainee doctor’s perception of self before entry into practice**

- How would you describe your performance in medical school?
- What kind of doctor would you ascribe to be?
- What kind of doctor would you hate to be?
- At this level, when someone calls you doctor, how do you respond? Why?

1. **Perception of self, based on external evaluation**

- What kind of positive feedback did you get from superiors, lecturers and peers while you were in medical school?
- Can you tell me how the feedback has motivated you to do better?

1. **Understanding expectations and preparedness for practice**

- How prepared are you for practice?
- What is it in your degree that makes you feel prepared?
- Can you tell me an important information you received in medical school that may be important during your medical internship?
- What aspect of practice do you think would be more challenging?
- Do you think your peers would face similar challenges or is this personal to you?
- Can you tell me about your experience in medical school?
- Can you talk me through how a typical day looked like in medical school?
- What does professionalism mean to you ?
- Can you describe any moments or experiences during your formal medical training that really represented what good medical professionalism is?
- Can you describe any moments or experiences during your formal medical training that you felt was not a show of professionalism?

**Questions and probes for medical officer interns**

Goal of interviews: Reflection of the journey of clinical practice so far and how this has changed their expectations.

1. **Opening/General question**

- Tell me about what you did while waiting to begin your internship.
- What personally attracted you to the medical profession?

1. **Understanding of internship**
2. How was it like moving from medical school to practice?
3. So far, how would you describe an internship in the Kenyan healthcare environment?
4. Can you describe what the first 3 weeks of the internship looked like?
5. Can you help me understand your work a little better, by perhaps describing what you do on a typical day?
6. **Professionalism**
7. Can you describe what you do on a typical day to help me understand your work a little better?
8. What does professionalism mean to you
9. Can you describe any moments or experiences during your medical training that really represented what good medical professionalism is?
10. Have you had to give up anything about yourself to be a good doctor?
11. From your experience, would you say that being a doctor is something you DO, or something you ARE?
12. **Relationship with mentors and supervisors**
13. How would you describe your relationship with other health workers (fellow doctors, clinical officers, nurses) so far
14. Were there any people who played a particularly significant (positive or negative) role during your internship?
15. During your internship, would you describe your relationship with your supervisor?
16. **Preparedness for practice**
17. Which discipline did you begin your internship ?
18. How well prepared do you feel medical school prepared you for internship and clinical practice?
19. What aspects of clinical practice did you feel you were particularly well prepared for?
    - Can you give me a situation where this knowledge was applied and what was the result?
20. -How did this make you feel as a doctor?
21. What aspects of your internship you believe you should have been better prepared for during medical school?
22. Can you describe the time when you needed this knowledge in practice?
23. How did this experience make you feel as a doctor?
24. What unexpected challenges did you face during your internship?
    - When was this? Can you describe the situation?
    - How did this make you feel as a doctor?
25. How did you deal with these challenges?
26. How will you describe the pressure to be creative in patient care. can you give an example of this? How did this make you feel as a doctor?
27. How has your experience of the internship affected your views of medicine and clinical practice?
28. Since you started your internship, have you ever thought about leaving the profession? (if yes, can you describe what happened around that time?
29. **Advise and next steps.**

Do you have any suggestions for incoming trainees in their preparation for internship? If so, what would it be?

How has the internship affected the way you see your medical career?

What are your plans moving forward?

# Sub-study C: Medical officers’ reflection of internship experiences and subsequent career transitions

**Questions (and probes) for medical officers:**

1. Please tell us about yourself (refer to the spreadsheet)
   1. What’s your current position? What type of occupation is it?
   2. Where did you complete your internship? Was it a public, private or mission hospital?
   3. Where did you undertake your undergraduate studies? Was it public or private?
2. How would you describe your internship experience?
   1. What are some of the things you have enjoyed most about your internship? Things least enjoyed? Can you give examples?
   2. Have you come across the term burnout, what does it mean to you? (state of emotional, physical and mental exhaustion caused by excessive and prolonged stress)
   3. How would you describe the level of support you received during internship? From whom?
   4. How do you think of patient safety in your internship hospital?
   5. How would you describe the level of preparation you got in medical school compared to the tasks you were given in your internship?
   6. How is it different from your intern friends who work in different hospitals?
   7. Did Covid impact your internship? health worker strike? government decentralization?
   8. What do you think can be done to improve your internship training and experience?
3. Can you tell me how did you choose to work in this current hospital/institution? What are the factors that influenced your decision to work here?
   1. Is this hospital/institution your first choice? Did you also apply to other jobs? Is it hard to find a job? How long did it take?
   2. Do you think there is a difference in terms of reputation of public/private/faith-based hospitals? How does that influence your decision?
   3. Did your preference for future career change during medical school and internship?
4. How did your internship experience influence your career decision?
   1. Do you prefer to work in certain hospitals or institutions because of your internship?
   2. How did your interaction with consultants/supervisors during the internship influenced your future plan?
   3. Did your previous relationship with your other colleagues during the internship hospital influenced your career decision?
   4. Are there any other social or political factors that influenced your career decision?
5. How safe do you feel about your current work environment, for you and for your patients?
   1. Your personal workload (any challenges accomplishing this?)
   2. In terms of the culture of safety in the workplace, and protocols if any (protective gear, waste disposal, essential work supplies and sundries, any changes with COVID-19)
   3. Adequacy of communication between colleagues for the care of patients (especially from senior to juniors, across professional boundaries e.g doctors to nurses)
   4. Teamwork (collaboration, cooperation, rather than competition)
   5. Participation in leadership decisions
   6. How and what would you like to see changed regarding safety at your workplace?

**Questions (and probes) for consultants:**

1. Please tell us about yourself
   1. What’s your current position? What specialty are you in?
   2. What hospital are you in? Is it a public, private or mission hospital?
   3. How long have you worked here?
   4. How many interns have you supervised? How many are you supervising now?
2. What do you think of the quality of the incoming interns? How well do you feel medical schools prepare interns for the job they have undertaken as an intern?
   1. What could be done to better prep students for internship?
   2. From your experience have you noticed any variations/differences in how well interns are prepared for their internship (i.e. Public/private universities or locally trained/those who trained abroad?)
   3. What should med schools do to better prepare students for their internship?
3. Do you think the interns are competent as a general medical officer after this one-year internship training?
   1. As a consultant/supervisor do you ensure interns have accomplished this?
   2. Do they have adequate opportunities to practice during the internship?
   3. How do you usually supervise and teach the interns?
   4. Do you receive any support from the hospital as a supervisor?
4. What do you hear about the experience of medical interns more generally?
   1. Some interns report high level of burnout and inadequate support received. Do you think that’s common? Can you give some example?
   2. How do you think the work conditions in this centre influence the learning experience of interns?
   3. What systems/structures are there to support interns during their internship? What types of support do you think should be provided to medical officer interns?
   4. Did Covid impact their internship? health worker strike? government decentralization?
   5. How was internship during your time? what is different now?
   6. What recommendations can you give to better prepare interns for their internship tasks?
5. How do you think interns decide on their future career after their internship? Why do you think interns choose to work in public/private/faith-based hospitals after they are registered and licensed?
   1. Why many interns opt for private practice?
   2. What do you think of the reputation of these hospitals?
   3. Is it hard for interns to find a job now? How long does it take to find a job?
   4. Are you involved in recruiting for this hospital? How does recruitment happen?
6. Can you broadly comment about safety at your current work environment, for you, your workers and for your patients?
   1. Your personal workload (any challenges accomplishing this?)
   2. In terms of the culture of safety in the workplace, and protocols if any (protective gear, waste disposal, essential work supplies and sundries, any changes with COVID-19)
   3. Adequacy of communication between colleagues for the care of patients (especially from senior to juniors, across professional boundaries e.g doctors to nurses)
   4. Teamwork (collaboration, cooperation, rather than competition)
   5. Participation in leadership decisions
   6. How and what would you like to see changed regarding safety at your workplace?
