# Supplementary material for: Examining liminality in professional practice, relational identities, and career prospects in resource-constrained health systems: Findings from an empirical study of medical and nurse interns in Kenya
Source: Soc Sci Med. Author manuscript; Available in PMC 2025 Jun 21. (PMC7617787; doi:10.1016/j.socscimed.2024.117226)
Supplement: Supplementary Material [file EMS206504-supplement-Supplementary_Material.zip › 1-s2.0-S0277953624006798-mmc2.docx]

**Table 2. Additional data and quotes**

| **First order aggregate thematic codes** | **Quotes** |
| --- | --- |
| - Unmet learning needs; Learning critical skills via alternative sources - Sudden expectation by existing workers and patients to be ‘full’ doctor/nurse. - First point of contact for critical cases/Treated like a robot/thrown into the deep | - *“If you are a BScN, you are just treated as a specialised nurse. So once you show up … they say we have a BScN here, station them at NBU. And maybe back then in college you were at NBU for only one week.” (Nurse intern 9)* - *“The reality of the internship, what happens in Kenya, other than the few days that the consultants normally come for the ward round…All other times an intern is supposed to be in the ward, every other patient that is been admitted, the intern admits the patient, does the management for the patient if there is an emergency to be handled an intern is called to do the same... basically an intern sees all the patients.” (Medical doctor 7)* |
| - Lack of basic equipment & resources for quality care delivery - Absence of direct supervision; ‘no one cares’; interns running the hospital | - *“A baby came in shock, now the hospital has no oxygen, we don’t have oxygen on the wall like Kenyatta (teaching hospital) has, and we don’t have nasal prongs like the ones I saw in Kenyatta. So they use nasogastric tubes to administer the oxygen, something that I’ve never even seen. I don’t even understand how that I would experience such a thing…there was a clinical officer intern who taking his bag to go home. I just pleaded with him, ‘please just don’t leave me alone, I can’t do this please’, and he helped me apparently. The MOs in the hospital are also not reliable …the MO doesn’t even answer his phone…it was very weird again for me.” (Nurse intern 07)* - *“I was under a lot of pressure. You are left alone with managing two wards - the male ward and the female ward. You are managing like one hundred and something patients. I almost lost my mind. The consultant will not understand the pressure that we (interns) are going through, the registrars, the MOs will not understand that. The only thing they understand, ‘an MOI is here to work’. I was under a lot of pressure and honestly I just had to see a psychiatrist.” (Medical intern 18)* |
| - Un-problematising the uncertain/unfamiliar - New learning; absorbing practical norms; Learning to accept sub-optimal conditions; it’s the system - Learning to improvise; opportunity to combine old and new knowledge | - *“It was what the internship is meant to be which is just very fast paced. You get a lot of hands-on experience managing patients. So, it's just we do a lot of calls. I don't know which word I can use to describe it but, it was just everything in one pot. So you're learning, you're getting tired and frustrated, you’re experiencing what real life medical systems are or what real life hospitals actually feel like, interacting with different kinds of patients, yeah all those things.” (Medical doctor 29)* - *“When you are an intern, you are just fresh from school… you don’t want shortcuts.… (then) you’re alone with 25 patients, you won’t be able to give them proper nursing care that you were taught. So, I think that is now why the shortcut thing normally comes. Because by the time you are used to it now, you learn the shortcuts and you are like so I can do this, oh so I can do this”. (Nurse intern 24)* |
| - Some knowledge from med/nurse school seems irrelevant/Feeling stuck, stranded - Increased anxiety/ stress/ pressure; fear of looking weak - Heightened blame on interns for mishaps/increased need to defend self | - *“(Interviewer: So there is bit of blaming on interns.) Yes, yes and that becomes an issue and then , it colors the outlook of the intern …the intern is sort of punished by being called for every, every little thing, like even things that are not really call worthy…your opinion is really disregarded on one extreme end… now you're made to run all around the hospital for things that are not even necessary just because someone made a mistake somewhere and they are trying to pin it on the intern.” (Medical doctor 30)* - *“I was in this scenario where I had like multiple casualties. Now the practice is to triage, see who you can attend to first… You have one ambulance that can refer one patient, and you have four patients that need to be referred. So you have to decide who goes first and when, and in what order… For an ambulance [to get to the referral hospital], it is like four hours one way … four hours to and from … so that one ambulance leaves and you know these remaining three people… you hadn’t envisioned you would ever lose someone but you know: ‘Well they are going to die and they shouldn’t die.’” (Medical intern 20)* |
| - Not good enough to be called a nurse/doctor, but no time to learn to be one - Expected to know everything as patient lives at risk - Intern instruction is disregarded but expected by other workers | - *“We usually just have that expectation of, ‘you are going to learn, you are going to be smarter than you were back in undergraduate’, you do not meet it at all. You are going to have; you just have to believe in yourself and work from the word go.” (Nurse intern - FGD1)* - *“When you step into that internship centre, you are not a student, you are a qualified nurse and that is how people see you.” (Nurse intern* *03 )* |
| - Overt conflict tension with workers in other cadre. Interns seen as “threats” - Existing worker intimidation based on status, age, length of experience/struggle to earn respect; ‘your degree is nothing’ (as a nurse from experienced diploma nurses) - Not treated with respect/ embarrassed by others; “internshit” | - *“These are people who have been there longer. They are older than you, more experienced than you but you're supposed to lead the team. How are you going to do that? How you're going to earn their respect? For me, it was very difficult to earn their respect” (Medical intern 01)* - *“I had a lot of trouble with other nurses, becausethey want to show you your degree is nothing and stuff like that. You should conform to what is on the ground and stop talking about things like ideals and stuff…As the situation on the ground makes you, it just makes you angry and you get frustrated.” (Nurse Intern 28)* |
| - Deliberate actions for collaborative work; pressure to present a united team - Humility, servanthood; listening leads to increased learning opportunities - Relinquish leadership/decision-making status; ‘I am not your boss’; ‘assign your selves’; treat other cadre as equals | - *“You always have to remember that despite being the leader, you should also remember that there are some things that you won’t know.…most of the time, an MO Intern will have had more theoretical knowledge in school, as compared to say the clinical officer interns. But then clinical officer interns will have more hands-on experience, as compared to medical officer interns. So, despite being the leader, you should remember that you still need them. You teach them and they will teach you. It is a two-way traffic relationship” (Medical intern 21)* - *“… patient rounds would involve every single person from the consultant to probably the medical officer or the resident…then to medical intern, then the clinical officers with clinical officer interns, including the nurses and the pharmacist. So, decisions that were made about patients were multidisciplinary in nature, and that meant that the standard of care would be high. A lot of things that probably that I never saw as a medical student I came to appreciate in [Hospital J].” (Medical doctor 04)* |
| - Emotional support from fellow interns (immediate and past); physical, psychological and mental relief - Forming empowering relationships with seniors and other cadre | - *“The kind of support I got mostly was from the senior MO interns, because they have gone through that journey. First of all, they help you settle because you have come in…So they're helping you look for a place to settle where you can be living, the houses that are available.…And then they also help you to settle in the hospital, in that they help introduce you to people so that you actually feel at home. And then after that they hold your hand now, in the practice of medicine in that they are going to help you know, this is how you make a diagnosis… So they walk you through that journey.” (Medical doctor 23)* - *“On my first day, I mentally crashed. I remember that at 3am, I called the other intern who was in the same department with me but had been there longer than I was…I completely crashed and went into a mind block. Thankfully, they came to meet me.” (Medical intern 31)* |
| - Anticipation/disappointment about anticipated internships - Anticipation/disappointment about doctor/nurse (stable professional) identity - Anticipation of professional confidence | - *“The expectations I had for my internship was really to put the knowledge and the skills I acquired as a student into practice in a broader perspective, because as an intern you have the opportunity and that space to do those things… at least now you have the autonomy and the space to do what you were unable to do as a student…. And then secondly of course, you see internship that is when you get to earn some stipend, so really my expectation was to get that stipend as well and probably start changing my life and getting to do other things after internship with that stipend.” (Nurse intern - FGD4)* - *“I wanted to actually major in anesthesia, I would have wanted a hospital that was ultramodern. A hospital that had a Cath lab, an ICU, probably HDU a place where you could actually practice anesthesia. But unfortunately… once we balloted I found out that the hospital that I wanted to go to I was not able to get to that hospital.” (Medical doctor 23)* |
| - Uncertainty of jobs; “tarmacking” future career - Uncertainty of specialization - Renewed hope for a future in the profession - Uncertainty of location to practice | - *“So after the three years I will have to go back to school because you cannot be a medical officer forever especially in the current terms of work of two or three years, you just have to have a plan B.” (Medical doctor 16)* - *“Now that there are no jobs in Kenya, thanks to devolution of health. When you finish, you start tarmacking (struggling to find a job) and pray to God to get employed. If you do, well and good if not, you tarmack until you do…. I am even thinking of going outside the country.” (Medical intern 11)* |
